# Supplementary material for: Morphological and Molecular Characterization of Some Alternaria Species Isolated from Tomato Fruits Concerning Mycotoxin Production and Polyketide Synthase Genes
Source: Plants (Basel). 2022 Apr 26;11(9):1168. doi: 10.3390/plants11091168 (PMC9103205; doi:10.3390/plants11091168)
Supplement: Supplementary file 1 [file plants-11-01168-s001.zip › plants-1632411-supplementary.pdf]

Figure S1. The most common *Alternaria* toxins.

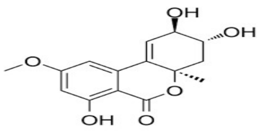 Altenuene	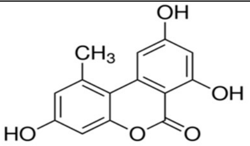 Alternariol	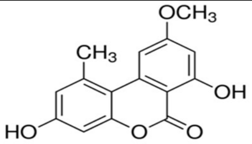 Alternariol methyl ether	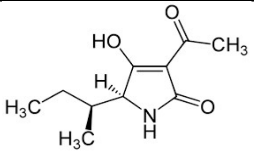 Tenuazonic acid

Figure S2. Tomato infected with *Alternaria*.

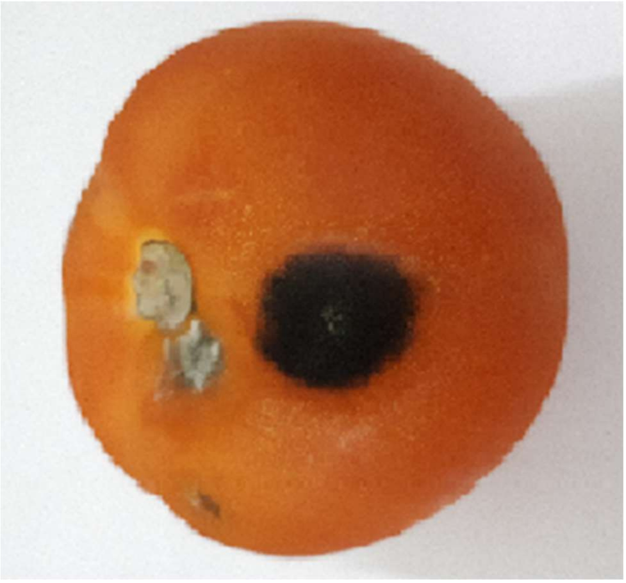

Table S1. Total counts, (calculated per (3x4) x 20 pieces), number of cases of isolation (NCI, out of 20), occurrence remarks (OR) and the relative importance value (RIV) of fungal genera and species isolated from tomato fruits.

Genera & Species	TC	%	NCI&OR	RIV
Alternaria	251	59.9	20 H	169.9
A.alternata	74	20.6	7 M	55.6
A.brasicicola	24	6.7	3 L	21.7
A.citri	72	20.1	6 M	50
A.radicina	49	13.6	6 M	43.6
A.tenussima	32	8.9	3 L	23.9
Aspergillus	17	4.7	3 L	19.7
A.fumigatus	1	0.3	1 R	5.3
A.niger	16	4.5	3 L	19.5
Cladosporium cladosporioides	32	8.9	4 L	28.9
Cochliobolus	24	6.7	7 L	41.7
C.lunatus	14	3.9	4 L	23.9
C.spicifer	10	2.8	4 L	22.8
Rhizopus stolonifer	1	0.3	1 R	5.3
Setosphaeria rostrata	10	2.8	4 L	22.8
Sterile mycelia (dark & white color)	4	1.1	2 L	11.1
Ulocladium alternariae	20	5.6	4 L	25.6
Gross total count	359			
Number of genera	7			
Number of species	13			

Occurrence remarks: OR (out of 20 samples) H=high occurrence from 10-20 cases, M= moderate occurrence from 5 – 9 cases, L = low occurrence from 2 – 4 cases and R = rare occurrence 1 case.
